# Warm Water Dressings

**Published:** 1863-10

**Authors:** Morton Monroe Eaton

**Affiliations:** Peoria, Illinois


### WARM WATER DRESSINGS.

By MORTON MONROE EATON, M. D.,

*Of Peoria, Illinois,*

I do not now recollect of ever having seen an article advocating warm water dressings for wounds. Why this is, I am at a loss to know. I expect that many surgeons of talent and large experience will be disposed to think my views on the treatment of wounds as erroneous, still I am not disposed to be frightened from advocating what I have proven to be the truth. True, I am a young man; and for this reason feel like shrinking from the stand I now fearlessly take, of advocating the use of warm water dressings; which I know will be looked upon by some uncharitably; but hope that those who may chance to read this monograph may be induced to test the truth of my assertions, knowing as some do that my opportunities for investigation, for a young man, have not been few, either in hospital, military or private practice, and have embraced all capital, as well as minor, operations.

In most of my first experience with wounds I used cold water dressings. In fact I was impressed with the idea that they were the best and absolutely necessary. I took notice, however, that the compresses that had been applied wet and cold, became very soon warm and were then a warm application. This called my attention to the benefit we derived from the cold in the first instance, which I must say, I could not see—and after watching cases closely, and reasoning from what is known of the reparative processes carried on by nature unaided, in the cure of simple injuries, noticing that every wound, if left to itself, will become more or less inflamed, the blood rushing to the surrounding tissues so far as vitality still exists, that in cases where we keep the parts very cold by the constant application of "cold water dressings," the healing process was often entirely arrested, and it often became necessary to apply some stimulating wash or liniment, to awaken more activity, and increase the circulation of blood, which we had been so eager to prevent at first, so as it was said, "to prevent inflammation." This showed plainly to my mind that a certain amount of warmth and redness, (often spoken of erroneously as an unhealthy condition,) was absolutely necessary to the most rapid healing of every wound—that keeping up the temperature of the parts to that of the natural standard in health, was desirable, and that as a rule, warmth was required rather than cold, and also that the exclusion of the atmospheric air is more essential than either; which, by the way, would of itself keep up the warmth in the parts quite efficiently in many cases. Therefore, I was convinced that the shock of a cold application to the wound from two to fifteen times a day, retarded the healing of the wounds, and often produced "inflammation," the very condition we professed to be guarding against.

The way I conceive this to be accomplished is this. The application of cold chills the parts and repels the blood that had filled the capillaries around the wound, puts a stop to the exudation of plastic material, and until the cold compress becomes warm the wound remains unchanged, and it takes sometimes an hour before the necessary warmth is re-established. Now again apply cold, and so on, at short intervals, and you may keep the wound from healing an indefinite period. Luckily it is not the practice to change cold dressings more than three or four times a day, as a rule, and from the time the compress becomes warm till it is again made cold reparation goes on.

Warm water dressings are especially applicable to gunshot wounds, as around these the tissues are deadened, often for the space of two or three inches. (This every army surgeon well knows.) So dead in fact, that if warmth is not soon applied the surrounding tissues slough off for a large space around. This is also the case with many cases of contused wounds from Railroad accidents, &c. In these cases, if warmth can be applied it is the best, as I have proven in numerous instances at the siege of Vicksburg, during the three weeks I was engaged with our unfortunate wounded there, and also in a number of instances which have occurred in this city during the last two years. True, it is not always practicable on the battle-field to apply warm water, then the next best thing should be done, apply cold water compress and direct that it shall not be changed or wet with cold water till the clóths are dry. By this means we have warmth most of the time. If inflammation of an angry nature should arise, a fomentation with hops is the best application. My own experience is that wounds will heal in about half the time with warm dressings that they will when applied cold and often renewed. I refer the reader to one of my operations reported in the Philadelphia *Med. and Sur. Reporter*, Vol. X, No. 3, in which I removed nearly the whole of the ilium in a severely comminuted state, the wound being extensively lacerated, with some of the fragments of bone thrust among the bowels, even down deep in the pelvis, which I dressed (after the first few minutes) with warm water dressings exclusively. The wound healed rapidly from the first. My patient suffered no inflammation in the bowels or peritoneum, and but slightly in the bladder. He walked around freely without aid, with no lameness, in three weeks, and the wound, which was as large as my fist, was entirely healed in four weeks, and he has never had any trouble from it since. This case was one that emphatically called for cold applications, according to the generally accepted views of their action. The situation of the wound—its contused and lacerated character—the extensive comminution of bone, as well as the patient's habits, (he being a confirmed drunkard,) all rendered the case liable to take on inflammatory action, but nothing of the kind occurred. 'Tis true the season of the year makes some difference; in warm weather the temperature of the water should be lower than in cold. The true object of the dressing should be borne in mind, which should be to maintain an equal temperature as nearly as possible somewhat above the natural heat of the body, and all sudden shocks by the application of cold should be avoided.

